# Sentiment Analysis of Comments of American Birders during Two Waves of the COVID-19 Pandemic Reveal More Negative Sentiments in the Context of Birding

**DOI:** 10.3390/ijerph182413142

**Published:** 2021-12-13

**Authors:** Christoph Randler, Nadine Kalb, Piotr Tryjanowski

**Affiliations:** 1 Department of Biology, Eberhard Karls Universität Tübingen, Auf der Morgenstelle 24, D-72076 Tübingen, Germany; nadine.kalb@uni-tuebingen.de; 2 Department of Zoology, Poznań University of Life Sciences, 60-625 Poznań, Poland; piotr.tryjanowski@gmail.com; 3 Department of Applied Geoinformatics and Spatial Planning, Czech University of Life Sciences Prague, Kamýcká 129, CZ-165 00 Prague 6, Czech Republic

**Keywords:** COVID-19, sentiment analysis, birdwatching, birding

## Abstract

Human–nature relationships are an important aspect of leisure research. Previous studies also reported that nature-related activities have a health benefit. In this study, we surveyed US-American birdwatchers at two time points during the COVID pandemic (independent samples). During the beginning of the COVID pandemic in spring 2020, we analyzed their comments with an AI sentiment analysis. Approximately one year later (winter 2020/21), during the second wave, the study was repeated, and a second data set was analyzed. Here we show that during the ongoing pandemic, the sentiments became more negative. This is an important result because it shows that despite the positive impact of nature on mental health, the sentiments become more negative in the enduring pandemic.

## 1. Introduction

In 2020, the outbreak of COVID-19 (Corona Virus Disease 2019) affected nearly all people’s daily lives, both at the workplace and during leisure activities [1,2,3]. This pandemic represents a substantial threat to health [4,5]. According to behavioral immune system [BIS] theory, people are expected to have more negative emotions and a negative cognitive assessment when facing potential health threats [6,7,8]. These behavioural changes are assumed to be mechanisms for self-protection in risky situations. Recent studies investigating the effect of COVID-19 on people’s mental health revealed that social media users indeed developed more negative sentiments after the COVID-19 Epidemic declaration [9,10,11].

In many countries, state-imposed restrictions such as lockdowns, working-from-home policies and closings of kindergarten and schools to curb the transmission of the virus had an impact on human–nature interactions as well as outdoor activities [12,13]. This has severe impacts on mental and physical health because outdoor leisure activities are known to provide important recreational ecosystem services such as stress relief [14] and an increased sense of wellbeing [15]. For example, Lindemann-Matthies and Matthies [16] experimentally reported a lowering of blood pressure levels with increasing biodiversity. Dallimer et al. [17] showed in a large survey study that greater biodiversity was significantly related to higher wellbeing. Recent studies during the pandemic showed that people in Burlington, Vermont, USA, increased their visitation rate to nature areas [18,19].

Birding, i.e., the identification of birds by visual and auditory cues, is one of the most popular nature-based recreation activities in the US [20]. In 2016, about 45.1 million people observed birds in the US. In total, 86 per cent of the observed birds around their home, whereas 36 per cent were away-from-home birders [21]. Birding is a leisure activity that also significantly contributes to scientific data collection [22]. An online questionnaire survey across 97 countries showed that birders watched birds more locally and in smaller groups and altered the time spent on birding compared to before the pandemic [23]. Birding was even recommended for psychiatrists and psychotherapists during the pandemic to impose an indirect effect on the health care personnel on their patients [24].

Here, we analyzed the comments of American birders in an online survey to investigate if and how their sentiments towards birding changed from the start of the COVID-19 pandemic in spring 2020 to about one year later in the winter season 2020/2021. The American birders are among the largest population of birders in the world [20]. According to previous sentiment studies of comments in social media, we expected birders to have more negative sentiments in 2021 than in 2020. Due to stay-at-home policies, most birders might not have been able to travel away from home to observe birds and could not attend birding festivals due to social distancing. Lastly, birders might have had less time to observe birds [23] due to home-schooling or home-office, which could lead to more negative sentiments in 2021. This hypothesis is supported by the strength of the COVID outbreak in the USA, which was stronger during the second wave when governmental measures were also more restricted (https://www.corona-in-zahlen.de/weltweit/vereinigte%20staaten, accessed on 6 October 2021).

The birding scene in the USA is highly developed, e.g., with the international platform eBird for data submission and keeping records, which also constitutes an important data collection for citizen science [25]. The Cornell Lab of Ornithology is an institution with a long tradition in ornithological research, e.g., project Feeder Watch, Nest Watch, Backyard Bird Count [26]. Yearly events include the Christmas Bird Count from the National Audubon Society, which was initiated in 1900 and is one of the longest-running citizen science projects in the world [27]. There are also numerous birding festivals and birdwatching fairs throughout the country.

## 2. Materials and Methods

We started the first survey on 30 March 2020 and continued until 2 May 2020. The surveys were prepared separately, according to different COVID-19 waves [5]. Participants were recruited via many channels, e.g., using announcements on the webpages of large bird and nature-related organizations. Mailing lists were used from many US-American federal, state or regional chapters. Scientific ornithological unions, societies and clubs were asked for participation by using postings on their websites or by distributing the link on their mailing lists. The sample addressed serious birders and not casual ones who only bird at their feeder without engagement in an organization. We also asked participants to distribute the questionnaire widely [snowball effect]. The questionnaire was hosted on the German SoSciSurvey server to fulfil the European Union’s data privacy rules. The questionnaire for 2020 asked three demographic questions (sex, gender, residence and one open-ended question: “Did your birding activity change during the Corona crisis? If so, please note how it changed”). In the second study, data were collected between 19 December 2020 and 16 April 2021 by using a larger questionnaire containing Likert-type questions [coded from 1–5; from 1 = fully disagree to 5 = fully agree] about COVID [about motivations and recreational specialization], some demographic questions and one open-ended question concerning the same content as in the previous survey. The additional questions will be used for a different analysis (≈35 questions). The open-ended question of this study was: “Here you can describe other changes in your birding activities due to the COVID-19 pandemic that we have not asked about so far (e.g., the adoption of new ways of birding)”. For this analysis, we compared the two open-ended questions in both survey waves. In 2020, 1950 participants wrote a comment in the questionnaire, whereas in 2021, only 370 left a comment. 2.2% of participants participated in both questionnaires.

We extracted the raw comments from the 2020 and 2021 questionnaires. First, we analysed the data to obtain the most frequently used words and to identify the most positive and negative words across all participants of the survey, i.e., comments were not analysed for each participant separately but across all received answers to the open-ended questions.

Raw comments contain unnecessary data, which is not required for analysis. Hence, we performed several steps of data pre-processing with the tm package [28] in R [29] to remove unnecessary information such as punctuations, numbers, special characters (i.e., @, /, ?, \) and stopwords ([e.g., the, at, and, is). Furthermore, we excluded comments from an analysis that only consisted of one word [i.e., yes, no, increased, less, none, nothing, and n/a], resulting in 1817 comments in 2020 and 361 in 2021 for analysis. We also conducted text-stemming, meaning that all words were merged to a common representation, i.e., the stem of a word. For example, the words “birds”, “birding” and “birder” were reduced to the word “bird”. After cleaning the data, we created a term-document matrix, i.e., a matrix where each column represents one word. This matrix was then used to analyse the most frequent words used in comments and to create word clouds. We further performed a sentiment analysis with the tidyverse [30] and tidytext [31] packages to find the most positive and negative words in the comments using the Bing lexicon.

In a second step, we analysed all comments separately to obtain a sentiment score for each individual participant, i.e., we calculated a sentiment score for each comment to take into account individual changes in birding among participants. Hence, we performed a sentiment analysis using the raw comments [excluding single-word answers]. We used the raw comments to avoid any loss of information due to text stemming as stemming might reduce words to a stem that cannot be recognised by the “afinn” lexicon, which can lead to a failure in assigning sentiment scores to a given word stem. Before analysis, all comments were tokenised, i.e., all comments were broken into single words called tokens. The tokens were then compared to the terms presented in the sentiment lexicon “afinn”. This lexicon assigns words with a score ranging from −5 to 5, with negative scores indicating negative and positive scores indicating positive sentiments. After the lexicon had assigned values to the available words of a comment, we calculated an overall sentiment score per comment. However, some comments (418 in 2020 and 123 in 2021) did not contain a word presented in the lexicon, and hence no sentiment score could be assigned. Finally, we performed a t-test to compare sentiment scores between 2020 and 2021.

## 3. Results

**Figure 1:**
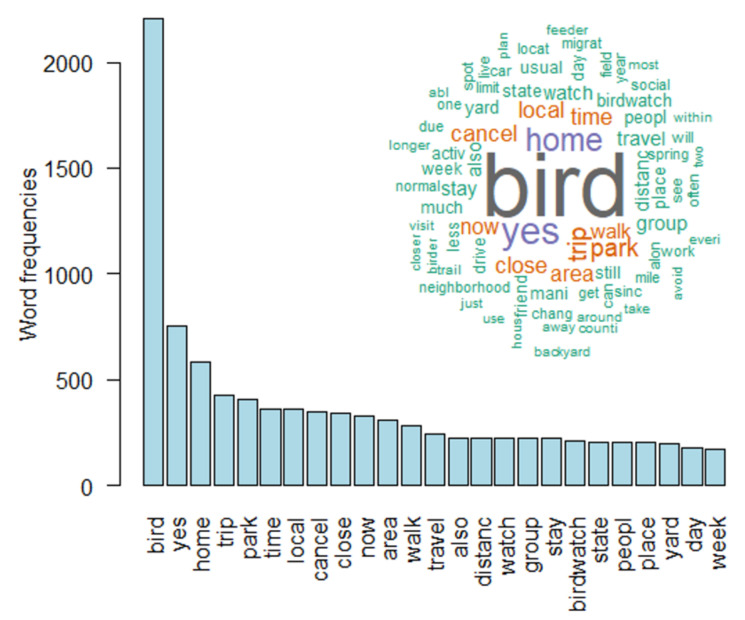
The bar plot illustrates the 25 most frequent words used in 2020 whereas the word cloud illustrates all words with a minimum frequency of 75.

**Figure 2:**
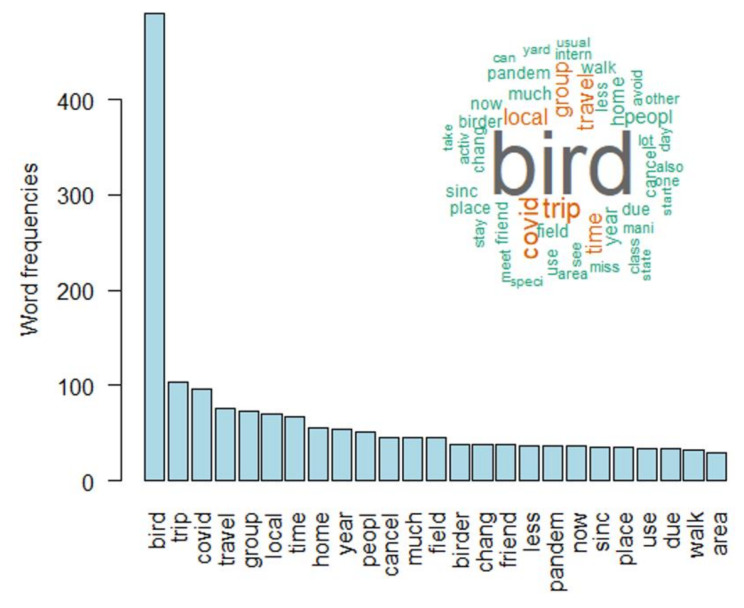
The bar plot illustrates the 25 most frequent words used in 2021 whereas the word cloud illustrates all words with a minimum frequency of 20.

Figure 1 and Figure 2 illustrate the most frequent words used in 2020 and 2021 across all comments after text stemming. In both years, “bird” was the most frequent term, followed by “yes” and “home” in 2020, “trip” and “COVID” in 2021.

The sentiment analysis revealed that in 2020 comments contained 488 negative polarity words and 688 positive polarity words, whereas, in 2021, comments contained 125 negative and 144 positive polarity words.

**Figure 3:**
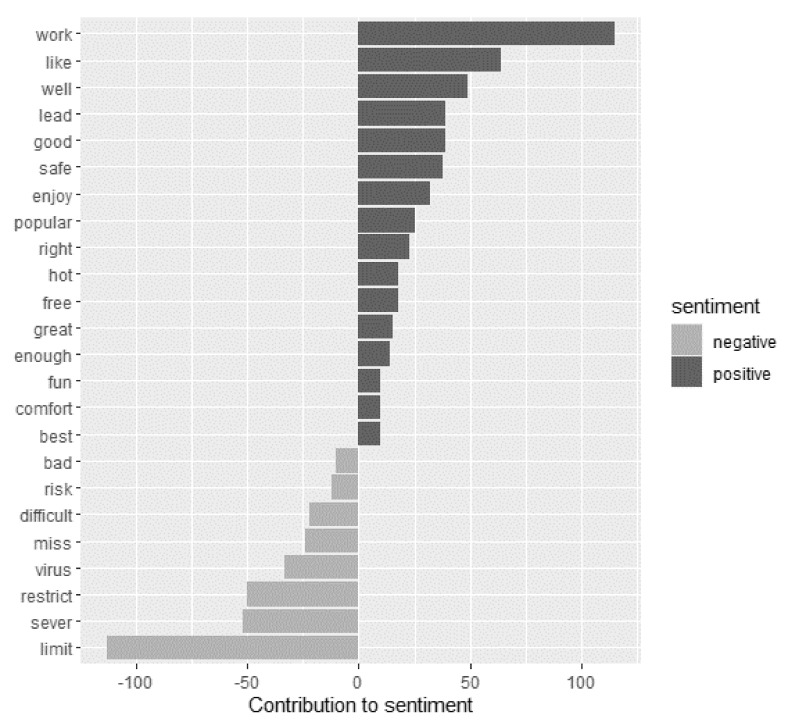
Words of the 2020 comments with the greatest contribution to the positive and negative sentiments. Words of all comments were compared to the terms presented in the sentiment lexicon “afinn”, which assigns scores from −5 to 5, with negative scores indicating negative and positive scores indicating positive sentiments.

**Figure 4:**
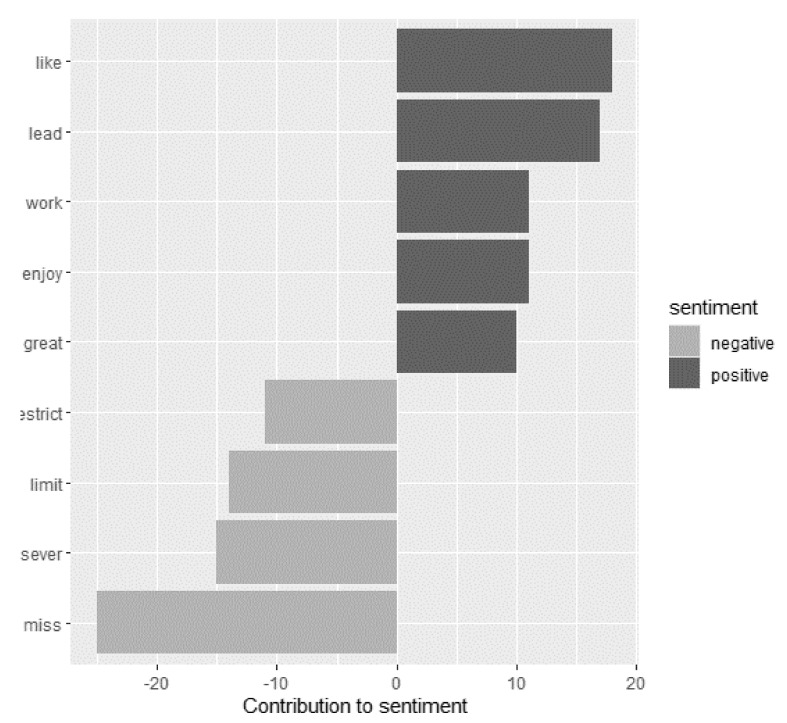
Words of the 2021 comments with the greatest contribution to the positive and negative sentiments. Words of all comments were compared to the terms presented in the sentiment lexicon “afinn”, which assigns scores from −5 to 5, with negative scores indicating negative and positive scores indicating positive sentiments.

The results of the sentiment analysis per comment without text stemming revealed a significant difference between sentiment scores of 2020 and 2021 (t = −2.97, df = 329, *p* = 0.003), whereby sentiment scores were more negative in 2021 (mean ± SD, −0.100 ± 2.331) than in 2020 (0.387 ± 2.409). In 2020 the words “work” (positive) and “limit” (negative) had the greatest contribution to the sentiments (Figure 3). In 2021 “like” (positive) and “miss” (negative) had the strongest impact on the sentiments (Figure 4).

Qualitative responses indicate some interesting aspects, e.g., considering the healthiness of birding (“found out birdwatching is healthy”), but also the frustration about cancelled field trips (“field trips have been cancelled. Now I have to go look for birds alone, and I am new at it and do not know many of the migrating species, so I get frustrated easily not being able to ask others about the birds I am seeing.”) or closed areas (“all my regular birding spots have been closed-beaches, state and county parks.”) as well as a restriction to areas near home (“I stay local, not travelling more than five miles.”).

## 4. Discussion

The ongoing pandemic has not only changed the work-life of most people but also how they spend their leisure time [2,32]. During a recent survey in the US, more participants reported an increase in outdoor activities than no change or a decreasing engagement in outdoor leisure activities [12]. Most activities that were of increasing popularity [e.g., gardening, hiking, jogging, walking, and watching wildlife] are easy to access and realize and can be conducted almost anywhere [12]. Bird watching is one of the outdoor leisure activities that increased during the COVID-19 pandemic [33,34]. In this study, we analyzed the free text comments of an online survey among birders asking whether COVID-19 affected their bird watching behavior. In 2020, the most frequent words were “bird”, “yes”, and “home”, indicating that many participants changed their birding activity (“yes”) and stayed more at home or watched birds closer to their homes. In 2021 the most frequent words shifted to “bird”, “trip” and “COVID”, indicating that many participants probably altered their birding trips (e.g., closer to home, fewer trips) due to social distancing than before the pandemic. This is in line with findings of a previous survey across various countries that birders watched birds in a smaller radius around their homes than before the pandemic [23]. Another interesting finding is that “COVID” or “corona” is not among the 25 most frequent words in 2020, whereas it is among the three most frequent words in 2021. In 2020, “corona” was mentioned 25 times, “COVID” 28 times and “coronavirus” 16 times. In 2021, in contrast, participants did not use the terms “corona” or “coronavirus” but mentioned “COVID” 96 times. Hence, participants altered their language as they used the correct term “COVID” instead of “corona” in 2021 and the higher frequency indicates that participants became more aware of COVID-19 over time as they received more information about the pandemic [35,36,37]. Our findings are in line with analyses of comments in social media showing that users altered their language during the course of the pandemic. For example, Weibo users, a popular online platform in China, used more words of concern after the outbreak of COVID-19 than before [9]. In addition, Twitter users in Wuhan, China and Lombardy, Italy showed changes in the frequency of word categories after lockdowns [38].

In addition to a change in word frequencies, we found birders to have more negative sentiments in 2021 than in 2020. This is not surprising as several studies found a shift towards more negative sentiments in headlines and social media comments after the COVID-19 outbreak in 2020 [9,11,39], even though also positive sentiments [e.g., associated with work-from-home policies and prevention managements] during this time have been reported [39,40,41,42]. A shift towards more negative emotions can be explained by the Behavioral Immune System [BIS] theory, which predicts people to have emotions that are more negative and a negative cognitive assessment when facing health threats [6,7,8]. These changes lead to a higher degree of self-protection against potential diseases as people tend to avoid infectious situations such as sick patients or social gatherings. In the case of birders, this most likely leads to a reduced groups size while birding or birding alone rather than in a group as reported in Randler et al. [23]. Moreover, birders could not attend bird festivals or travel to distant places to observe rare species during the pandemic, which could also lead to more negative sentiments in 2021. Rice et al. [43] showed that especially residents of urban areas decreased their frequency of outdoor recreation activities as well as the distance travelled to such activities more than participants living in rural areas. Moreover, birders seem to have stayed closer to home to observe birds during the pandemic than before [23,33]. This trend is supported by an analysis of data submitted to the citizen science platform iNaturalist, where birders can submit their [daily] bird observations. Here, urban observations increased during the lockdown in Italy and Spain, compared to previous years, whereas the daily number of non-urban observations decreased [33]. A similar pattern was also reported for the platform eBird as observations in urbanized regions increased in April 2020 compared to previous years, whereas observations submitted from rarer wetland habitats were less prevalent [44]. The lockdown itself may have had a negative effect on birders, but not necessarily for birds because the birds changed their land use [45].

The two data collections were made at different times during the year (seasons: 30 March–2 May 2020 and 19 December 2020–16 April 2021), which might affect the birdwatchers’ answers. Birdwatchers, at least in the northern parts, such as Alaska, are usually more active during the bird migrating seasons and summer, but not in winter. As the USA is a large country, birder behavior may differ between the northern states, where birding during winter may be less important or rewarding (but see exceptions such Snowy Owls, *Bubo scandiacus*, in Central Park, NY, USA) compared to the southern states where the weather might be better, and wintering migrants are present. Therefore, if birders were not very active in their birding during the second wave of data collection, their comments might come out more negative or not at all.

The sample is restricted to birders that have at least some serious interest, and absolute novices or casual birders who are not integrated into local chapters, organizations, etc., were not addressed in this study. Confirmed birders are perhaps those who suffered the most from the pandemic in their practice, while the successive lockdowns may have allowed novices to pay more attention to nature in their immediate environment. It would be interesting to assess if and how laypeople may have started birding throughout the pandemic and whether they sustain in this activity for a longer time. However, this can be only addressed in a representative population study.

However, despite the limitations that the two questions were not identical and that time of data collection was not equivalent within the same season, we feel that this study makes a valuable contribution above and beyond the existing literature on sentiment analysis because it addressed a target population in two different waves which makes it valuable in comparison to other analyses, e.g., on Twitter or Facebook, where most analyses are based on the comments without knowing a little bit about the population studied.

Both studies differed in the response rates towards the open-ended questions, but we can only speculate about this. This is comparable to other sentiment analyses where standardization is often not possible.

## 5. Conclusions

In summary, our results suggest that even though the frequency of bird watching has increased during the pandemic [23], other aspects of birding, including travelling to special places to see rare species, attending birding festivals, and birding in groups, has changed, which most likely led to a shift towards more negative sentiments during the pandemic. Especially highly specialized birders, i.e., birders with a high level of commitment [46], are more likely to feel restricted in their birding activities by travel restrictions than less involved birders. Hence, future studies might also investigate if and how the specialization of birders affect the sentiments towards birding during the COVID-19 pandemic. Also, an interesting point would be to study novices that began birding during the lockdown and may have experienced this activity as a relief.

## Data Availability

Data will be uploaded to the Open Science Framework after acceptance.
